# A Proposed New Model to Explain the Role of Low Dose Non-DNA Targeted Radiation Exposure in Chronic Fatigue and Immune Dysfunction Syndrome

**DOI:** 10.3390/ijms24076022

**Published:** 2023-03-23

**Authors:** Alan Cocchetto, Colin Seymour, Carmel Mothersill

**Affiliations:** 1 National CFIDS Foundation Inc., Hull, MA 02045-1602, USA; 2 Department of Biology, McMaster University, Hamilton, ON L8S 4K1, Canada

**Keywords:** CFIDS/ME/CFS, ionizing radiation, UVA, reactive oxygen species, systems biology, melanoma, hematopoietic dysregulation

## Abstract

Chronic Fatigue and Immune Dysfunction Syndrome (CFIDS) is considered to be a multidimensional illness whose etiology is unknown. However, reports from Chernobyl, as well as those from the United States, have revealed an association between radiation exposure and the development of CFIDS. As such, we present an expanded model using a systems biology approach to explain the etiology of CFIDS as it relates to this cohort of patients. This paper proposes an integrated model with ionizing radiation as a suggested trigger for CFIDS mediated through UVA induction and biophoton generation inside the body resulting from radiation-induced bystander effects (RIBE). Evidence in support of this approach has been organized into a systems view linking CFIDS illness markers with the initiating events, in this case, low-dose radiation exposure. This results in the formation of reactive oxygen species (ROS) as well as important immunologic and other downstream effects. Furthermore, the model implicates melanoma and subsequent hematopoietic dysregulation in this underlying process. Through the identification of this association with melanoma, clinical medicine, including dermatology, hematology, and oncology, can now begin to apply its expansive knowledge base to provide new treatment options for an illness that has had few effective treatments.

## 1. Introduction

### 1.1. Background to the Model

CFIDS is known by several names that include Myalgic Encephalomyelitis (ME) as well as Chronic Fatigue Syndrome (CFS) [1]. Previous reports from Chernobyl and the United States have associated radiation exposure with CFIDS [2,3,4,5,6,7,8]. One report had identified chromosomal damage in a CFIDS patient cohort that had previously been screened for the presence of urinary radionuclides [9,10].

**Figure 1:**
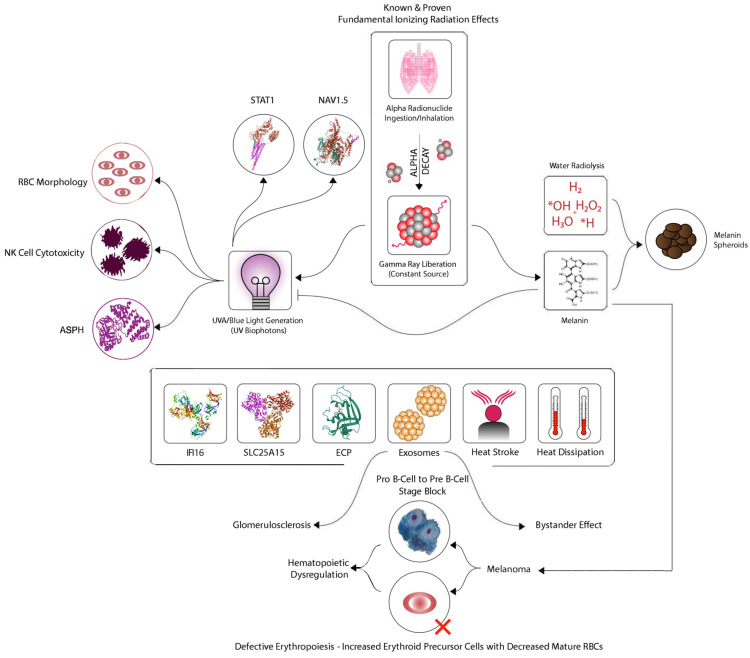
The proposed model: In this CFIDS model, internal radionuclide exposure generates gamma-rays to produce UVA biophotons. Various biomarkers are shown that are common to both CFIDS and melanoma. Hematopoietic dysregulation is a key characteristic in this process. The various steps in the model including the chemical (redox) reactions are described in the text.

In this new systems biology model for CFIDS (Figure 1), the overall hypothesis is that exposure to radionuclides, either via ingestion or inhalation, leads to the constant liberation of low-level electromagnetic radiation due to radioactive decay. This could be viewed as a pathological process that develops gradually as a result of constant and repeated exposure to low doses of radiation, where localized internal damage would accumulate over time. Such an accumulation would be dependent on rates of radionuclide decay as well as the body’s ability to deal with such an exposure by depuration, damage repair, or other mechanisms [11,12].

### 1.2. Background to the Proposed Mechanism

Cell exposure to beta or gamma rays has been shown to generate UVA and blue light biophotons [13,14]. The emission of light from irradiated organic material has been documented since the 1930s [15]. More recently our group characterized the emissions using a single photon counter and showed that the emitted light is mostly in the UVA range and low wavelength blue light [16,17]. The light emission can be shown to increase with radiation dose, and the biological response varies depending on the p53 status of the cell line that is exposed to the biophotons [18]. Investigations of the mechanism revealed that photosensitizers enhanced the biological cell-killing effect of the photons while melanin suppressed it [17,19]. Further examination of the system suggested that mitochondrial function was implicated, and a study of the electron transport chain confirmed that the function of mitochondrial complex 1 was completely blocked in cells receiving photon signals from other cells exposed to ionizing radiation [20]. Taken together, this body of work proves that cells grown in media containing radioisotopes or exposed to external radiation emit biophotons. These biophotons are mainly in the UVA wavelength band, and they can trigger downstream stress-like responses in cells receiving the biophotons that were never exposed to ionizing radiation. The involvement of mitochondrial complex 1 in the mechanism strongly suggests that ATP depletion and the consequent elevation of reactive oxygen species (see below) are key components in compromising efficient cell function. The mechanism is considered to explain bystander signaling, which is a non-targeted effect where cells exposed to ionizing radiation signals in non-irradiated cells induce responses indistinguishable from those seen in directly irradiated cells [15].

### 1.3. The Bridge from Bystander Signaling Stress to CFIDS

Human skin contains 64% water, while water makes up 70% of a cell’s volume [21]. When water interacts with radiation, known as water radiolysis, it undergoes decomposition to produce hydrogen radicals, hydrogen peroxide, and other assorted oxygen compounds [22]. Thus, the net effect of water radiolysis is the formation of free radicals or reactive oxygen species (ROS), which are representative of cellular, tissue, or organ-based oxidative stress when applied to human biology. In CFIDS patients, blood parameters, indicative of oxidative stress, are associated with patient symptom expression [23]. These data suggest that oxidative stress, due to excess free radical formation, may be a contributor to the pathology of CFIDS.

Ultraviolet (UV) radiation exposure is normally considered a “skin first” or “outside-in” model since solar radiation, located exterior to the body, is familiar to those humans who have experienced a sunburn. Since UV radiation is considered a human carcinogen and mutagen, it is not surprising that UV radiation exposure from sunlight has both short-term as well as long-term negative effects. In brief, the short-term or acute effects of UVB on normal skin include inflammation or erythema due to sunburn and subsequent tanning, while UVA mainly acts by causing oxidative stress and increases in ROS, both of which lead to increased risk of DNA damage, bystander effects, and genomic instability [24,25,26,27,28]. Long-term or chronic effects of UVA and UVB lead to immunosuppression and DNA damage, which produce changes in the skin itself as well as various forms of skin cancer, including melanoma [29,30]. The biological impact of oxidative stress due to UVA radiation exposure includes lipid peroxidation, protein carbonylation, mitochondrial DNA (mtDNA) deletions, immunosuppression, immune modulation, cellular apoptosis, and changes to cellular defense as well as energy metabolism [31]. Many of these elements exist within the currently known framework of CFIDS.

Since our exposure model considers internal radionuclide deposition, this leads to the “blood first” or an “inside-out” characterization of this process. Because beta and gamma radiation cell exposure has been shown to generate UVA photons [17,19], it is important to consider its potential durational characteristics. For example, the alpha-radionuclide uranium has a very long half-life. As such, alpha-particle decay would be expected to liberate gamma radiation for a long time as well. The dose amount and exposure timeline may affect this. Potentially, these characteristics would impact UVA photon liberation levels and duration, thus representing chronic UVA photon cellular exposure. This would affect cells, tissues, and organs. Since the alpha-particle is either ingested or inhaled, the “inside-out” model applies due to the fact that this radiation source is internal. Some radionuclides are known to ‘home’ to the bone, threatening the marrow; for example, radium which competes with calcium. This becomes a key point in this part of the model, especially in light of melanoma characteristics.

### 1.4. The Connection to Melanoma

The pathogenesis of UVA radiation-induced melanoma is complex but includes factors such as unrepaired DNA damage, which leads to mutagenesis, and immunosuppression that help to drive melanomagenesis [32]. A critical element adding to the importance of this model is the fact that protection from UV light is an evolutionarily conserved feature of the hematopoietic niche [33]. In this paper, hematopoietic stem and progenitor cells (HSPCs) operate within a specific microenvironment, referred to as the hematopoietic niche, to regulate HSPC behavior. The research referred to was conducted on zebrafish, where the blood-forming organ is in the anterior kidney and not the bone marrow. The authors found that a layer of melanocytes protects the kidney cells, as they have no bone protection. The authors suggest that melanocytes above the stem cell niche protect HSPCs against UV light-induced DNA damage. This niche, however, is located in the bone marrow of adult mammals, where the bone protects the HSPCs. The principle of melanocytes protecting HSPCs is important to note, as this research may help to yield new ideas regarding the development of melanoma and its subsequent treatment. Of critical importance, it has been found that melanoma-induced immunosuppression is mediated by hematopoietic dysregulation [34]. This dysregulation by melanoma induces both a “Pro B-cell to Pre B-cell stage block” as well as “defective erythropoiesis via increased erythroid precursor cells with decreased mature RBCs.” Thus, melanoma causes both a disordered B-cell population as well as defective erythropoiesis to develop. In addition, Pro B-cells become Pre B-cells when they express membrane mu chains with surrogate light chains in the Pre B-cell receptor [35].

### 1.5. CFIDS and Cancers Other Than Melanoma

In light of the B-cell block noted above, a study by the National Cancer Institute found that CFIDS was associated with a significantly increased risk of non-Hodgkin lymphoma (NHL) [36]. Among NHL subtypes, CFIDS was associated with diffuse large B-cell lymphoma, marginal zone lymphoma, and B-cell NHL not otherwise specified. CFIDS associations with NHL overall and NHL subtypes remained elevated after excluding patients with medical conditions related to CFIDS or NHL, such as autoimmune conditions.

CFIDS was also associated with cancers of the pancreas, kidney, breast, oral cavity, and pharynx in this study. Initial clinical observations on CFIDS suggesting its association with B-cell lymphomas date to early reports from the Lake Tahoe outbreak [37]. In addition to lymphomas and a lack of B-cells reported in patients, kappa-lambda light chain clonal abnormalities were identified in 25% of fifty CFIDS patients. Furthermore, another study identified that 94% of the CFIDS patients evaluated had a B-cell immunodeficiency with a marked depletion of their CD19 + sIgM + mature B-lymphocyte population [38]. These mature B-cells ranged from a low of 0.5% to a high of 53% in patients. In addition, this study also identified a T-cell immunodeficiency in 26% of patients. Thus, a high incidence of severe B-cell immunodeficiency in CFIDS patients was identified.

It is important to note that gamma radiation has also been found to alter the oxidation–reduction behavior of melanin, which results in electric current production [39]. This research adds to the understanding of the interactions between ionizing radiation and melanin.

Lastly, additional support for this model comes from a substantial study that was undertaken by the Swiss government [40]. In this study, researchers looked at the effects of both radon, an alpha-radionuclide, as well as UV radiation exposure on malignant melanoma (MM) mortality.

Switzerland has among the highest mortality rates for MM in Europe. The incidence of MM in both men and women in Switzerland has more than doubled in the last 20 years. This study included 5.2 million adults. The main model considered MM as the definitive primary cause of death. Interestingly, while natural UV levels are relatively high owing to the elevation in the alpine regions, certain areas of Switzerland have elevated radon levels because of the underlying geology, which leads to high doses of radon. In this large cohort, this study found a statistically significant increased risk of death from MM and skin cancer in general in adults associated with exposure to radon, and therefore this study was found to support the hypothesis that radon exposure is a relevant risk factor for skin cancer independent of residential UV exposure.

## 2. Various Biomarkers Tied to UVA Exposure and to Melanoma

In this section of the paper, the evidence linking various biochemical pathways and markers to CFIDS etiology is presented. Knowledge of these mechanisms may help to ultimately impact therapeutic treatment.

### 2.1. STAT1

The STAT1 protein is a critical cell protein for proper immune function and regulation. Without it, cells are unresponsive to interferons, thereby leaving the body defenseless against viral and bacterial infections. Previous research has shown that a subpopulation of CFIDS patients who have a STAT1 deficiency exist [41,42]. This immunodeficiency may underlie the increased susceptibility to infections seen in many patients.

In our systems model, a biphasic pattern similar to that of STAT1 activity was observed as a function of the UVA radiation dose [43]. Low-dose UVA radiation was found to directly affect STAT1 phosphorylation. Thus, low-dose UVA radiation was found to activate STAT1, while higher-dose UVA radiation suppressed STAT1. Other research groups have found UV radiation to inhibit STAT1 [44,45]. Another group developed a novel radiation-biomarker discovery platform that represented the top 500 genes identified by linear regression analysis. This platform was then reduced to a 10-hub network that included STAT1 as a significant radiation target [46]. In addition, STAT1 was found to be strongly associated with overall survival in melanoma patients, where high STAT1 mRNA levels were associated with better survival outcomes [47].

### 2.2. NaV1.5

Ciguatoxins are a class of toxic polyether compounds found in fish whose consumption causes ciguatera poisoning [48]. Chronic ciguatera poisoning has been associated with CFIDS [49]. Ciguatoxins act on the neuronal voltage-dependent sodium (Na) channel NaV1.5 [50,51]. Dr. Yoshitsugi Hokama’s monoclonal antibody for CTX (Mab-CTX) directly detects alterations to NaV1.5 [52] and this specific antibody was previously identified to react with CFIDS patient blood as reported in several studies [53,54].

NaV1.5 is an integral membrane protein involved in the initiation and conduction of action potentials. Alterations to NaV1.5 have been associated with a variety of arrhythmic disorders, including long QT, Brugada, and sick sinus syndromes, as well as progressive cardiac conduction defect and atrial standstill. Changes in the NaV1.5 expression level and/or sodium current density have been frequently noticed in acquired cardiac disorders such as heart failure.

In our systems model, UVA radiation was found to hamper the fast inactivation of cardiac NaV1.5 [55]. Furthermore, the authors suggest that UVA radiation modification of NaV1.5 provides valuable clues for ischemia/reperfusion injury in the heart and the central nervous system. In addition, NaV1.5 has been found to be expressed in human melanoma cells and has been associated with cancer invasiveness and metastasis [56,57].

### 2.3. ASPH

ASPH, or asparaginyl beta-hydroxylase, has been found to be increased in CFIDS patients [58]. ASPH is a transmembrane protein and a member of the alpha-ketoglutarate-dependent dioxygenase family [59]. In the last few decades, accumulating evidence has indicated that ASPH expression is upregulated in numerous types of human malignant cancer and is associated with poor survival and prognosis [60]. The ASPH protein aggregates on the surface of tumor cells. ASPH is highly expressed in cancers of the liver, pancreas, stomach, colon, breast, prostate, lung and brain. ASPH is necessary and sufficient to promote tumor cell migration, invasion, motility, and distant metastatic spread both in-vitro and in-vivo [61].

In our systems model, UVA radiation was found to significantly upregulate ASPH [62]. ASPH may prove to be a novel immunotherapy target for patients with melanoma [60].

### 2.4. NK Cell Cytotoxicity

NK cells, also known as natural killer cells, are a type of cytotoxic lymphocyte critical to the immune system. NK cells have the ability to recognize and kill cells in the absence of antibodies and the major histocompatibility complex, thereby allowing for a quicker immune reaction to stressed cells. NK cells and B-cell lineage differentiation derive from a common lymphomyeloid hematopoietic progenitor [63]. NK cell cytotoxicity has previously been shown to be altered in CFIDS patients, where it can impact the cell’s functionality [64]. Various NK cell subsets have been previously evaluated to uncover their degree of radiation sensitivity [65]. This group identified a highly pronounced decrease in the CD3-CD8+CD56+ bright subset of NK cells after radiation exposure. Furthermore, this subpopulation was found to be the most radiosensitive one. Interestingly, three CFIDS patient studies have each identified a decrease in CD3-CD8+CD56+ bright NK cells in these patient cohorts [66,67,68].

In our systems model, previous studies have shown that NK cell activity is suppressed by UVA radiation, which results in the suppression of delayed hypersensitivity responses and thus impacts immunity [69,70]. Furthermore, circulating CD56 bright NK cells inversely correlates with the survival of melanoma patients [71].

### 2.5. RBC Morphology

Changes to red blood cell rheology or shape have been previously identified in CFIDS patients [72,73,74]. Patient samples were found to lack deformability, indicated by the presence of stomatocytes or other non-discocytic surface changes on red blood cells (RBCs). RBC deformability is important for proper tissue perfusion and oxygenation due to the impact on microcirculatory blood flow.

In our systems model, previous research has shown that RBC rheology mainly depends on the spectrin network, which can be altered by oxidation processes within the cell [75]. This group used atomic force microscopy to study the changes in the spectrin matrix and RBC morphology during oxidation processes caused by UV radiation exposure. The number of normal discocytic RBCs decreased from 98% to 12% while generating increased numbers of stomatocytes, echinocytes, and spherocytes. Thus, the spectrin network was damaged by UV radiation exposure thereby adversely affecting RBC rheology and many of its downstream effects.

### 2.6. IFI16

IFI16, also known as gamma-interferon-inducible protein 16, is a sensor for intracellular DNA and a mediator of interferon induction as well as an innate antiviral defense. It was found to be significantly upregulated in CFIDS patients [76,77]. In parallel with an increased frequency of plasmablasts in CFIDS patients with relatively short disease duration, the expression level of IFI16 was found to be negatively correlated with disease duration in this cohort. In addition, IFI16 expression showed a positive correlation with IGHV3-30–3 frequency in CFIDS patients.

In our systems model, one group demonstrated that IFI16, normally restricted to the nucleus, could be induced to appear in the cytoplasm under conditions of UV radiation-induced cell injury [78]. Furthermore, IFI16 has been shown to be a novel signature associated with overall survival and immune infiltration of skin cutaneous melanoma [79].

### 2.7. SLC25A15

In a large UK Biobank CFIDS study, SLC25A15 was identified as being statistically significant. SLC25A15 encodes the Ornithine Transporter type 1 protein that transports ornithine across the inner membrane of mitochondria to the mitochondrial matrix and plays a role in the urea cycle [80]. This group suggested that SLC25A15 could be a causal gene for altered CFIDS risk.

In our systems model, one group identified that the overexpression of SLC25A15 was involved in the proliferation of cutaneous melanoma, leading to a poor prognosis [81].

### 2.8. ECP

ECP, also known as eosinophilic cationic protein, is a basic secretion protein involved in the immune system response [82]. ECP levels are an indicator of eosinophil-specific activation and degranulation. ECP levels have been found to be significantly higher in CFIDS patients and are modulated by exercise challenge [83,84].

In our systems model, ECP levels appear to be a novel prognostic serum marker for the overall survival outcome of melanoma patients [85]. Here, ECP levels were found to be inversely correlated with survival.

### 2.9. Heat Stroke and Heat Dissipation

In CFIDS patients, increased temperature has been found in widely distributed regions of the brain [86]. This group reasoned that regional brain temperature has been used as a proxy for measuring neuroinflammation, with the observation that microglia activation can increase metabolic demands, potentially leading to excess heat. Interestingly, another research group had outlined the similarity in the pathophysiological mechanisms that apply to heat stroke and overlap with CFIDS [87]. According to this group, the endotoxemia pathway is increasingly considered the leading driver of severe organ damage and the main cause of death in people suffering from heat stroke.

More recently, it was reported that Transient Receptor Potential Melastatin 3 (TRPM3) activity was lost in CFIDS patients, and there was no significant difference in TRPM3 ion channel activity between CFIDS patients and post-COVID-19 patients [88]. Interestingly, TRPM3 functions as a sensor for noxious heat and underlies heat sensitivity in a subset of sensory neurons [89]. In fact, TRPM3-deficient mice exhibited clear deficits in their avoidance responses to noxious heat and in the development of inflammatory heat hyperalgesia. Heat stroke and heat dissipation, in the context of melanoma, are both involved in thermoregulation. This is in line with transient receptor potential ion channels and their role in both thermoregulation and thermosensation [90].

In our systems model, heat plays an additional role in melanoma. One group utilized thermal conductivity measurements as a tool to detect the micro-invasion of melanoma [91]. In accordance with tumor progression, effective thermal conductivity was higher in invasive melanoma. Likewise, another research group had identified fever as a factor contributing to long-term survival in a patient with metastatic melanoma [92]. They presented the unique case of a female patient who had suffered from MM for more than 13 years. The patient had several episodes of fever that were not deliberately treated with medication. After each fever episode, the patient observed the disappearance of tumors, which was confirmed by medical examination. Interestingly, since her initial diagnosis, the patient has refused most of the proposed medical treatments. Most of her malignant tumors have either disappeared or stabilized without further growth.

### 2.10. Exosomes

Exosomes are small vesicles enclosed by a lipid membrane bilayer and secreted by most cells in the body. They have been shown by our group to be released in response to the UVA bystander signal [16]. The treatment of unirradiated cells with these exosomes alone triggers a radiation-induced bystander effect, thus neatly confirming that the UVA signal triggers exosome release and may be upstream of exosomes in the mechanism of the radiation-induced bystander effect (RIBE) [93]. MicroRNAs (miRNAs) are the most numerous cargo molecules in the exosome. Because numerous miRNAs have been identified to date in CFIDS patients, we have chosen to focus on one in particular because of its importance to this model. One group has reported an increase in miR-21 in both moderately ill as well as severely ill CFIDS patients’ plasma and peripheral blood mononuclear cells (PBMCs) [94]. This result was confirmed in different patient cohorts. According to this group, miR-21 down-regulates the Sirt1/eNOS axis via TGF-beta and TNF-alpha pathways in endothelial cells as well as endothelial progenitor cells. However, another research group reported a significant reduction in the expression levels of miR-21 in both the NK and CD8 T-cells in CFIDS patients [95].

Among the key research findings that support our proposed systems model is that miR-21 has been identified as being intimately involved in RIBE. Significant upregulation of miR-21 was found by Xu [96] in both directly irradiated cells and bystander cells, which was confirmed by the expression of miR-21 precursor and its target genes. Additional research has generated a proposed RIBE model for exosome-mediated transfer of miR-21 [97]. Thus, in irradiated cells, the expression of miR-21 is upregulated, and as a response, miR-21 sorting to exosomes is motivated. The exosomes are secreted out from the irradiated cells, diffused into the extracellular medium, and taken up by non-irradiated cells. The miR-21 inside the exosomes is then released into bystander cells to induce bystander effects. Once the exosome cargo, including miR-21, was released into the cytoplasm of recipient or bystander cells, the increased miR-21 level regulated the relevant target gene expression and induced chromosome aberration and DNA damage. In addition, miR-21 has been found to play a key role in melanomagenesis and melanoma progression [98]. Lastly, miR-21 has been found in glomerular injury as well as glomerulosclerosis [99,100].

## 3. Discussion

To assess and compare other potential CFIDS models to the systems model presented herein, we first performed numerous searches, in Pubmed, using the following query terms—Chronic Fatigue and Immune Dysfunction Syndrome disease model, Chronic Fatigue Syndrome disease model, as well as Myalgic Encephalomyelitis disease model. In addition, we reviewed several well-known research-based books on the subject as well as past issues of the Journal of Chronic Fatigue Syndrome [101,102,103]. Our intent was to examine the literature for any human pre-existing hierarchical disease models that utilized an integrated macroscopic-microscopic approach for the creation of a potential CFIDS model.

One such model, created by Englebienne and DeMeirleir, discussed how different onset mechanisms and changes to the immune system could elicit a number of events and symptoms that would not spontaneously reverse [102]. In their model, the authors discuss the patient onset and/or predispositional factors, such as viral or bacterial infections, that negatively impact the immune system. These bring about various intracellular changes, such as altered apoptosis, that trigger various biological events, such as T-cell activation and cytokine storm, to ultimately lead to patient symptoms, such as pain and malaise.

From our literature search, we learned that most current CFIDS papers focused on identifying various diagnostic markers or discussed limited functional mechanisms associated with the disease. Given that CFIDS is considered to be a complex multisystem disease that appears to be heterogeneous as a result of its case definition, this has created a tremendous challenge for those researchers and clinicians alike who are left to study and subsequently treat the disease. However, we do believe that multiple patient cohorts may exist that satisfy the current CFIDS case definition and that our system model, which points to intimate involvement of melanoma in some patients, may ultimately prove to be a reflection of this. Furthermore, the general consensus is that CFIDS is an immunological disease. In this paper, NK cell cytotoxicity, STAT1, and IFI16 are examples of this. Melanoma is not only one of the most immunogenic cancers but also one of the most effective cancers at subverting host immunity. This may prove to be a critical crossroad, from an immunological standpoint, that requires much greater scientific effort to gain a true understanding and appreciation for this part of the disease process. As such, additional CFIDS patient research confirming and delineating these mechanisms may be in order especially given the cancer patient mortality associated with melanoma outcome in those patients whose detection is delayed. Melanoma’s propensity to metastasize makes early recognition and excision the most important factor in patient survival [104].

Since our system’s model is based on internal radiation exposure, several additional important comments should be taken into consideration. The National Research Council publication Health Effects of Exposure to Low Levels of Ionizing Radiation (BEIR V) states that “the skin has a higher susceptibility to radiation carcinogenesis than has generally been suspected” and, while proposing risk estimates for both basal cell and squamous cell carcinomas, does not mention melanoma [105].

An extensive review of melanoma and ionizing radiation was previously generated by Fink and Bates [106]. Here, the authors examined data from the Canadian Radiation Dose Registry, nuclear industry workers, subjects near nuclear test blasts, survivors of the atomic bombings of Japan, airline pilots and cabin attendants, recipients of medical radiation, and radiological technicians. The authors provided evidence for elevated risks for melanoma related to exposure to ionizing radiation. The 2017 Swiss government-based paper on radon exposure and melanoma, mentioned earlier, is in line with the 2005 paper by Fink and Bates on ionizing radiation exposure and increased melanoma risk. Furthermore, in a large 29,000+ human study of the effects of external radiation exposure on the mortality of French nuclear workers, among the twenty-one main cancer sites studied, a statistically significant cancer excess was observed only for skin melanoma [107].

Given the possible elevated risk for CFIDS patients to develop melanoma, we suggest the following clinical testing be considered. The first would be to utilize increased skin cancer screenings to improve active surveillance for potential dermatological skin changes in CFIDS patients. Any skin changes should be closely monitored so as to minimize long term patient risk for melanoma. Secondly, to utilize the quantitative measurement of Eosinophil Cationic Protein (ECP) in human serum. This test is commercially available in the United States, and it may be available elsewhere. As mentioned previously, ECP has been found to be significantly elevated in CFIDS patients, and since it is a prognostic serum marker for melanoma, we encourage its use in these patients. Furthermore, obtaining a baseline measurement in a patient may prove to be a useful addition to a clinician’s diagnostic arsenal as it may assist in monitoring potential disease progression. As such, we firmly believe that these clinical tests may prove to be helpful in CFIDS patients who may be at potential risk for melanoma development.

The fact that the mechanisms described here involve a continuous exposure to stress such as UVA radiation, biophotons, radionuclides, etc. suggests that an accelerated aging process, rather than an increased cancer risk, may be associated with CFIDS. Conversely, some literature data suggest that, to potentially lead to a cancer progression, the exposure to stress should be repeated, chronic but with some periods of non-stress to permit cells to propagate errors and to bypass cell cycle arrests. In this regard, research at the CDC has identified premature telomere attrition in CFIDS patients [108]. The telomere length was shorter, and this translated to roughly 10+ years of additional aging in patients. Telomeres, which act as molecular caps at the ends of chromosomes to protect humans against aging and cancer, have been shown to have a surprising inability to protect themselves against UV radiation [109,110,111]. Thus, the chromosome’s guardians have been found to be susceptible to UV radiation effects, and the damage to telomeres was not repaired. As telomeres shorten, cells age, deteriorate, and eventually die. As cells divide over a lifetime, telomeres tend to wear down, and the resulting chromosomal instability can potentially lead to an increased cancer risk. Increasing evidence has been gathered that shows that the long-term effects of radiation exposure are due to oxidative changes leading to the continuous accumulation of DNA damage in the progeny of both irradiated and non-irradiated bystander cells and that telomeres are a key player in radiation-induced carcinogenesis [112]. According to a more recent paper by Samuel, ergothioneine has been shown to mitigate telomere shortening under oxidative stress conditions [113]. As such, we suggest additional research, both at the bench and in a clinical setting, to determine the potential utility of the use of L-ergothioneine as a treatment option for CFIDS patients.

Overall, the mechanism that we have proposed here integrates UVA radiation, biophotons, and radionuclides, whose molecular responses obey different kinetics according to the specific DNA damage they induce. For example, the repair of DNA base damage induced by UVA radiation is expected to be faster than that of DNA strand breaks induced by radionuclides. Our question going forward is: how do we best integrate these features into the model as well as the clinical picture in CFIDS patients?

## 4. Conclusions

This paper presents a new model using systems biology to integrate information from the literature across several systems and several levels of organization to produce a new fundamental model to explain CFIDS etiology, which potentially connects to melanoma development. Through continuous internal radiation exposure caused by internal emitters of ionizing radiation, UV biophotons are liberated. While the model focuses on ionizing radiation due to ingested or inhaled radioactive particles as an example of a possible causative factor for CFIDS, this methodology could also apply to other trigger stressors where elevation of ROS is involved in the disease process. The benefits of this systems model approach are that it helps identify key points in the mechanism where targeted treatment interventions could be possible. Some of these, such as the use of melanin and L-ergothioneine, are actively being researched by our group [16,114,115,116,117,118].

## Data Availability

Not applicable.
